# Investigation of the occupational exposure to blood-borne pathogens of staff at a third-class specialist hospital in 2015–2018: a retrospective study

**DOI:** 10.1038/s41598-022-05436-z

**Published:** 2022-01-27

**Authors:** Yuanyi Ji, Junbo Huang, Guoguo Jiang, Qiaolan Liu, Dalei Xiao, Jianjun Deng

**Affiliations:** 1 Nosocomial Infection Management Department, West China School of Public Health and West China Fourth Hospital, Sichuan University, Chengdu, Sichuan People’s Republic of China; 2 Nosocomial Infection Management Department, Chengdu Second People’s Hospital, Chengdu, Sichuan People’s Republic of China; 3 Department of Health-Related Social and Behavioral Sciences, West China School of Public Health and West China Fourth Hospital, Sichuan University, Chengdu, Sichuan People’s Republic of China; 4 Nosocomial Infection Management Department, West China Second University Hospital; Zigong Maternal and Child Health Hospital, Sichuan University, Chengdu, Sichuan People’s Republic of China

**Keywords:** Infectious diseases, Disease prevention, Preventive medicine, Health occupations, Risk factors

## Abstract

To understand the current situation of occupational exposure to blood-borne pathogens in a women's and children's hospital and analyze the causes to provide a scientific basis for improving occupational exposure prevention and control measures. We analyzed occupational exposure to blood-borne pathogens in a third-class women's and children's hospital from 2015 to 2018, considering the workers’ occupational categories and length of service; the sites, types, and causes of exposure; and the pathogens of the source patients. From 2015 to 2018, there were 146 cases of occupational exposure to blood-borne pathogens, mainly from sharp-instrument injuries (81.5%; 119/146). Trainees represented the highest proportion of occupational exposure (30.1%; 44/146), followed by nurses (29.5%; 43/146). Occupational exposure among staff with less than one year of service accounted for 43.2% (63/146) of cases. Fisher's exact test showed that different occupational groups had different types of occupational exposure, and among the occupationally exposed populations, the proportion of sharp injuries is higher than that of blood and body fluid exposure, and the difference is statistically significant (*χ*^2^ = 12.937, *P* = 0.008). Different occupational groups faced exposure to different types of pathogens: medical staff were more likely than workmen to be exposed to hepatitis B, while workmen were more likely than medical staff to be exposed to unknown pathogens; these differences were statistically significant (*χ*^2^ = 55.344, *P* < 0.001). Health records were established for all cases of occupational exposure to blood-borne pathogens, and no staff members contracted a blood-borne disease due to occupational exposure. In order to reduce occupational exposure, regular training in occupational protection for junior medical staff and workers should be strengthened, the monitoring and protection system of occupational exposure to blood-borne pathogens improved, standard prevention measures strengthened, operations standardized, safe injection equipment provided, and comprehensive measures taken.

## Introduction

Occupational exposure to blood-borne pathogens refers to the state in which workers encounter blood or other potentially infectious substances containing blood-borne pathogens through the eyes, mouth, nose or other mucous membranes; damaged skin; or parenteral routes during occupational activities^1^. Occupational exposure has always been one of the main problems that medical staff suffer from occupational-related blood-borne pathogen infections, and strong preventive measures have been taken all over the world^2^. In the United States, the Centers for Disease Control and Prevention (CDC) has proposed its preventive measures nationwide, and since the mid-1980s, when medical staff report HBV, especially HIV, they have provided guidelines. The hospital was required to prepare a hard system to discard sharps and needles contaminated with blood, and to keep official records of related injuries. In Japan, people have realized that they are facing a large number of needles stick injury, and occupational exposure to blood-borne pathogens is also one of the serious problems in medical care^3–5^. In the current medical environment in China, hospital staff members are faced with a limited number of doctors relative to the number of patients, an excessive workload and strained doctor-patient relationships and are often in a state of intense activity and high risk^6,7^. In the event of occupational exposure, the physical and mental health of medical staff could be severely impaired. The "Guidelines for the Prevention of Occupational Exposure to Blood-borne Pathogens", proposed in 2009, and the "Occupational Disease Classification and Catalogue", revised in 2013 to include AIDS among medical personnel due to occupational exposure, indicate the state’s increasing attention to the occupational exposure of medical personnel^1,7^. Therefore, to fully understand the current situation of occupational exposure to blood-borne pathogens among the staff at a women’s and children’s hospital, reduce the risk of such exposure, and improve monitoring and protection against this threat, the present study retrospectively analyzed 146 cases of occupational exposure in a women's and children's hospital from 2015 to 2018.

## Methods

### Source

This study examined a total of 146 cases of occupational exposure to blood-borne pathogens that occurred in a third-class women's and children's hospital from January 2015 to December 2018. The inclusion criteria are: (1) it occurred within the scope of the hospital; (2) the exposure was the staff of the hospital; (3) needle stick injury or skin and mucous membrane exposure occurred. The exclusion criteria are: the key information is incomplete and cannot be supplemented.

### Methods and statistical analysis

Data on exposure to blood-borne pathogens in 2015–2018 were collected from medical personnel occupational exposure case registration forms, which include basic personal information, occupational category, location, type, cause, source of pathogens, emergency treatment after exposure and post exposure prophylaxis; follow-up surveys were conducted for each case of occupational exposure. The informed written consent was obtained from all participants. The ethics approval of this study was obtained from Medical Ethical Committee of West China School of Public Health and West China Fourth University Hospital that approved the research, confirm that all research was performed in accordance with relevant guidelines/regulations.

The medical staff occupational exposure case registration form was uniformly coded; data were entered using *EpiData 3.1* software, and data collation, statistical description and statistical analysis were performed using *SPSS 20.0*. The data were statistically described by rates or composition ratios. Comparisons between the data groups were performed by the *χ*^2^ test or Fisher’s exact test, and the significance level was *α* = 0.05.

### Explanation of proper nouns

(1) Workmen: The workers in our hospital include cleaners, porter, central transportation workers, plumbers, etc. (2) Interns: The definition of “intern” is an intern doctor, an intern nurse, a regular training doctor, and a regular training nurse. (3) Third-class specialist hospital: In the classification of China's medical institutions, hospitals have been reviewed and divided into one, two, and three levels, and each level is subdivided into A, B, and C. Our hospital is a specialized hospital for a women’s and children’s hospital, and has been rated as a tertiary a level specialized hospital. (4) Scalp-vein needles: The scalp vein needle cannula includes a hollow tube. The front end of the hollow tube can be tightly sealed with the puncture needle. The side of the hollow tube is equipped with a flat needle handle, which can be left in the vein of the patient. It has a simple structure and convenient operation. In the case of no infusion, the characteristics of no hindrance to the patient.

### Ethics approval and consent to participate

The medical staff provided verbal and written consent to participate and the ethics approval of this study was obtained from Medical Ethical Committee of Sichuan University, China (No. 20140307).

## Results

Table 1 describes the profile of the sample group. There were 146 cases of occupational exposure to blood-borne pathogens in 2015–2018, involving 23 men (15.8%) and 123 women (84.2%). The largest occupational category consisted of internship trainees and trainers, accounting for 30.1% (44/146), followed by nurses, accounting for 29.5% (43/146). Less experienced workers represented more cases of blood-borne occupational exposure; workers with less than 5 years of tenure accounted for 71.2% (104/146) of the total number of cases, and workers with less than one year of tenure accounted for 43.2% (63/146).

**Table 1:** Basic situation of the occupational exposure of hospital staff to blood-borne pathogens, 2015–2018.

Variable	N (%)
**Sex**	
Man	23/146 (15.8)
Woman	123/146 (84.2)
**Occupational category**	
Clinician	24/146 (16.4)
Nurse	43/146 (29.5)
Medical technician	4/146 (2.7)
workmen	31/146 (21.2)
Internship trainee or trainer	44/146 (30.1)
**Length of service**	
≤ 1 year	63/146 (43.2)
1 ~ ≤ 5 years	41/146 (28.1)
5 ~ ≤ 10 years	23/146 (15.8)
> 10 years	19/146 (13.0)
**Types of occupational exposure**	
Syringe needle	47/146 (32.2)
Scalp needle	21/146 (14.4)
Suture needle	23/146 (15.8)
Scalpel	10/146 (6.8)
Other sharps	18/146 (12.3)
Exposure of blood and body fluids to skin and mucosa	27/146 (18.5)
**Pathogen species**	
Negative	25/146 (17.1)
Hepatitis B	45/146 (30.8)
Hepatitis C	3/146 (2.1)
HIV/AIDS	11/146 (7.5)
Syphilis	9/146 (6.2)
Unknown pathogen	53/146 (36.3)
**Exposure time**	
During surgery (suture/incision)	29/146 (19.8)
Stabbed by improperly placed sharp objects after operation	14/146 (9.6)
Examination, treatment, and other nursing activities after operation	14/146 (9.6)
During disposal of sharp objects	11/146 (7.5)
Handling the trans-shipment of medical waste	11/146 (7.5)
Venous puncture	10/146 (6.8)
Needle removal, needle separation and syringe at the end of the infusion	10/146 (6.8)
Discarding sharp objects such as needles	9/146 (6.2)
Cooperating with other personnel	6/146 (4.1)
Transferring sharps	4/146 (2.7)

Table 1 describes the types of occupational exposure to blood-borne pathogens among the hospital staff. Of the 146 cases, 119 involved sharp injuries, accounting for 81.5%, and 27 were blood fluid exposures, accounting for 18.5%. The sharp injuries included 47 cases involving syringe needles, 21 involving scalp-vein needles, 23 involving suture needles, 10 involving scalpels, and 18 involving other sharps. Table 1 presents the timing of blood-borne occupational exposure. These events were concentrated during operations; during examination, treatment, and other nursing activities after operation; after the completion of treatment; and during disposal of waste. The most common time of exposure was during surgery (sewing/cutting) (19.8%). Exposure also occurred due to sharp injuries after operations, either when staff members were stabbed by an improperly placed sharp object (9.6%) or when nurses were treating and checking on patients (9.6%). Occupational exposure occurred mainly in the ward (28.1%) and the operating room (26.7%).

The occupational exposure data of the 146 cases (Table 1) showed that the main blood-borne infection responsible for occupational exposure among hospital staff was hepatitis B (30.8%), followed by HIV/AIDS (7.5%); in another 36.3% of cases, the pathogen status of the source patient was uncertain.

Fisher's exact test (Table 2) showed that different groups sustained different types of occupational exposure. Among the occupationally exposed populations, the proportion of sharp injuries is higher than that of blood and body fluid exposure, and the difference is statistically significant (*χ*^2^ = 12.937, *P* = 0.008). The types of occupational exposure and pathogens also differed across occupational groups. Most medical workers exposed to occupational infection were exposed to hepatitis B, whereas workmen were mainly exposed to unknown pathogens. The difference was statistically significant (*χ*^2^ = 55.344, *P* < 0.001) (Table 3).

**Table 2:** Occupational exposure analysis of staff with different work experience and occupational category.

Variable	Types		Pearson *χ*^2^	*P-*value
	Sharp injury N (%)	Exposure to blood and body fluids		
**Occupational category**				
Clinicians	17/24 (70.8)	7/24 (29.2)	12.937	0.008
Nurse	30/43 (69.8)	13/43 (30.2)		
Medical technicians	3/4 (75.0)	1/4 (25.0)		
workmen	30/31 (96.8)	1/31 (3.2)		
Internship trainee or trainer	39/44 (88.6)	5/44 (11.4)		
**Length of service**				
≤ 1 year	53/63 (84.1)	10/63 (15.9)	5.835	0.115
1 ~ ≤ 5 years	18/20 (90.0)	2/20 (10.0)		
5 ~ ≤ 10 years	19/21 (90.5)	2/21 (9.5)		
> 10 years	29/42 (69.0)	13/42 (31.0)

**Table 3:** Analysis of the pathogens workers with different work experiences and occupational categories were exposed to (N = 146).

Variable	Exposure pathogens						Pearson *χ*^2^	*P-*value
	Negative	Hepatitis B	Hepatitis C	HIV/AIDS	Syphilis	Unknown pathogen		
**Occupational category**								
Clinician	1/24 (4.2)	12/24 (50.0)	0/24 (0.0)	5/24 (20.8)	2/24 (8.3)	4/24 (16.7)	55.227	< 0.001
Nurse	6/43 (14.0)	16/43 (37.2)	3/43 (7.0)	4/43 (9.3)	2/43 (4.7)	12/43 (27.9)		
Medical technician	0/4 (0.0)	1/4 (25.0)	0/4 (0.0)	0/4 (0.0)	1/4 (25)	2/4 (50.0)		
Workmen	9/29 (29.0)	0/29 (0.0)	0/29 (0.0)	0/29 (0.0)	0/29 (0.0)	22/29 (71.0)		
Internship trainee or trainer	9/44 (20.5)	16/44 (36.4)	0/44 (0.0)	2/44 (4.5)	4/44 (9.1)	13/44 (29.5)		
**Length of service**								
≤ 1 year	11/63 (17.5)	21/63 (33.3)	0/63 (0.0)	3/63 (4.8)	6/63 (9.5)	22/63 (34.9)	16.777	0.428
1 ~ ≤ 5 years	7/41 (17.1)	11/41 (26.8)	2/41 (4.9)	2/41 (4.9)	0/41 (0.0)	19/41 (46.3)		
5 ~ ≤ 10 years	3/23 (13.0)	7/23 (30.4)	1/23 (4.3)	3/23 (13.0)	3/23 (13.0)	6/23 (26.1)		
> 10 years	4/19 (21.1)	6/19 (31.6)	0/19 (0.0)	3/19 (15.8)	0/19 (0.0)	6/19 (31.6)		

Of the 146 individuals who experienced occupational exposure to blood-borne pathogens, 135 (92.5%) were subjected to emergency treatment: If the mucous membranes such as eyes are exposed, rinse with saline; If it is a sharp injury, extrusion, rinsing, disinfection, and dressing after exposure. All exposed persons underwent extensive immunological testing (including tests for hepatitis B, hepatitis C, HIV and syphilis), which can be used as a baseline result after exposure, and risk assessment was carried out based on the source patient's pathogen, the nature of the exposure, the staff member’s autoimmune status, and rational preventive medication. Occupational exposure health records were established for all cases of occupational exposure to blood-borne pathogens, and the exposed staff members were followed up regularly; no staff members were infected with blood-borne diseases due to occupational exposure.

## Discussion

### High-risk groups for occupational exposure

Between 2015 and 2018, a total of 146 cases of occupational exposure to blood-borne pathogens occurred in the hospital. Among the 146 cases who experienced occupational exposure, interns, internship trainees, trainers and nurses accounted for the highest proportion, which is similar to the results of Chinese and international research^7–11^. Most of the staff members involved in the clinical diagnosis and treatment process are involved in frontline work. Medical staff who are in internship training and regular training spend a relatively short time in the hospital. These staff are relatively unfamiliar with the hospital environment and equipment and are especially prone to occupational exposure. Teaching hospitals also receive more interns and training personnel than other hospitals do^12^. In addition, the survey found that less experienced staff members represented a high proportion of blood-borne occupational exposure cases, which was related to their relatively low attentiveness, skill levels, and awareness of self-protection measures^13^. Pre-employment and on-the-job training for less experienced staff and intern trainees should be strengthened. Different occupational categories had different types of occupational exposures, and the differences were statistically significant. Nurses represent a higher proportion of blood fluid exposure cases than other types of medical staff, suggesting that different intervention strategies should be targeted to different occupational categories to reduce the risk of occupational exposure. Notably, workmen face the third highest risk of occupational exposure, suggesting a high risk of occupational exposure among that group, and workers who need to focus on and training are those involved in the collection and disposal of medical waste^14^.

### Exposure type and time

Concerning the type of occupational exposure, sharp injuries accounted for the most cases. The sharps that cause sharp injuries are mainly syringe needles, scalp-vein needles, suture needles and scalpels, which is consistent with the results of Cho E^15,16^. Exposures occur mainly during operations (treatment, examination, surgery) and the handling and disposal of waste, and sharp injuries are especially likely to occur during invasive operations and operations with time constraints^17^. Often, sharp objects that are no longer being used and that have been improperly placed are involved in cases of exposure. This misplacement may be related to the specifications and convenience of sharps boxes. Sharps boxes should have different specifications and be convenient to use, and they should be placed within arm’s reach and in the field of view^18^. It is recommended that hospital staff strictly implement standardized prevention and operation processes.

### Source disease analysis

The investigation showed that the main blood-borne infection to which the hospital staff members were exposed was hepatitis B, as found in other studies^19–21^. China is a country with a high incidence of hepatitis B relative to the global level. The most effective way to protect workers from occupational exposure to the hepatitis B virus is vaccination against hepatitis B. Therefore, the immunization of hospital staff must be improved, with the aim of full coverage. Notably, the pathogens of the source patients involved in 36.3% of the occupational exposure cases were unclear. Most of the cases where the status of the patient is unknown are like this: Workers collect packaged medical waste in public areas such as medical waste storage rooms, hospital halls, and outpatient clinics. In this case, it is difficult to find specific patient information. In addition, according to Fisher's exact test, different occupational groups faced different types of infection from occupational exposure, and the difference was statistically significant. Medical staff were more likely than workmen to be exposed to hepatitis B, and workmen were exposed mainly to unknown pathogens. Workmen and medical staff are both high-risk groups; their awareness of occupational protection should be strengthened, and the process for handling medical waste should be standardized.

## Conclusions

### Occupational exposure protection strategy

The control of blood-borne infectious diseases should follow the principle of priority in the prevention and treatment of occupational diseases. The first is to eliminate risks, followed by engineering control, management measures and behavior control, and the third is personal protection and post-contact preventive measures.

1. Improve the hospital occupational protection management system and clarify the responsibilities of all relevant departments. Hospitals should establish and provide timely updates to the occupational exposure protection measures and treatment procedures for blood-borne pathogens in ways that correspond to the actual conditions at the hospital to protect the health and rights of hospital staff^22^.
2. Carry out diverse and targeted vocational training and education, such as pre-employment training, on-the-job training, special education, emergency drills, daily department meetings, daily in-house teleconferences and online training and assessment. Each department should participate in a medical quality assessment, and the safety awareness of all types of staff should be improved; the entire hospital staff should be included in these efforts. Moreover, each department should be conveniently equipped with personal protective equipment and sharps boxes according to its occupational exposure risks.
3. Provide safe working environments, equipment and supplies, such as safe injection equipment and various types of sharps, to reduce the risk of occupational exposure. It is recommended to use a needle-free system and a sharp object with a protective device^23^.
4. Implement an immunization system and inoculate staff against hepatitis B to prevent hepatitis B infection through occupational exposure. Each hospital staff member should be vaccinated according to his or her own immunization conditions.
5. Use behavioral changes to reduce the high-risk behaviors of medical staff, for example, by prohibiting the reuse of trocar caps, ensuring that sharps boxes are never more than 3/4 full and remain closed, strengthening preventive measures, and standardizing operation protocols.
6. Improve the monitoring and protection system for occupational exposure to blood-borne pathogens and establish occupational exposure health records for each hospital staff member to reduce the risk of occupational exposure.

## Data Availability

The datasets used and/or analyzed during the current study are available from the corresponding author on reasonable request.

## References

[1] Zhang M (2010). "Guidelines for the Protection of Occupational Contact of Bloodborne Pathogens" and its interpretation. Chin. Nurs. Manag..

[2] Cheetham S, Ngo HTT, Liira J (2021). Education and training for preventing sharps injuries and splash exposures in healthcare workers (review). Cochrane Database Syst. Rev..

[3] Tarigan LH, Cifuentes M, Quinn M (2015). Prevention of needle-stick injuries in healthcare facilities: a meta-analysis. Infect. Control Hosp. Epidemiol..

[4] Saadeh R, Khairallah K, Abozeid H (2020). Needle stick and sharp injuries among healthcare workers: A retrospective six-year study. Sultan Qaboos Univ. Med. J..

[5] Sivić S, Gavran L, Baručija A (2020). Epidemiological characteristics of accidental needle-stick injury among health care professionals in primary healthcare in Zenica. Med. Glas (Zenica).

[6] Wang C, Huang L, Li J (2019). Relationship between psychosocial working conditions, stress perception, and needle-stick injury among healthcare workers in Shanghai. BMC Public Health.

[7] Sun J, Xu H, Gu AM (2016). Investigation and analysis of occupational exposure and protection of medical staff in China. Chin. J. Infect. Control.

[8] Xiao Q (2014). The latest classification and catalogue of occupational diseases in 2014. Saf. Health.

[9] Ran SS (2017). Study on current status and preventive strategy of occupational exposure in a women and children’s hospital during 2011–2015. J. Mod. Med. Health.

[10] Różańska A, Szczypta A, Baran M (2014). Healthcare workers' occupational exposure to bloodborne pathogens: a 5-year observation in selected hospitals of the Małopolska province. Int. J. Occup. Med. Environ. Health.

[11] Kassa G, Selenic D, Lahuerta M (2016). Occupational exposure to bloodborne pathogens among health care workers in Botswana: Reporting and utilization of postexposure prophylaxis. Am. J. Infect. Control..

[12] Wang YJ, Meng ZH, Zheng XF (2015). The status of occupational blood and infectious body fluids exposure China: A 5-year review. Transfus Med..

[13] Wang YT, Qiao F, Huang WZ (2017). Analysis of sharp instrument injuries among healthcare staff in a comprehensive teaching hospital from 2011 to 2014. West China Med. J..

[14] Henk F, Van der Molen A, Koos AH (2011). Better effect of the use of a needle safety device in combination with an interactive workshop to prevent needle stick injuries. Saf. Sci..

[15] Rvbacki M, Piekarska A, Wiszniewska M (2013). Work safety among Polish health care workers in respect of exposure to bloodborne pathogens. Med. Pr..

[16] Cho E, Lee H, Choi M (2013). Factors associated with needlestick and sharp injuries among hospital nurses: A cross-sectional questionnaire survey. Int. J. Nurs. Stud..

[17] Bakaeen F, Awad S, Albo D (2006). Epidemiology of exposure to blood borne pathogens on a surgical service. Am. J. Surg..

[18] Hutin Y, Hauri A, Chiarello L (2003). Best infection control practices for intradermal, subcutaneous, and intramuscular needle injection. Bull. World Health Orqan..

[19] Deuffic-Burban S, Delarocque-Astagneau E, Abiteboul D (2011). Blood-borne viruses in health care workers: prevention and management. J. Clin. Virol..

[20] Wang J. *Occupational Exposure Analysis and Control Strategy of Blood-Borne Pathogens in Medical Staff—A Case Study of a Third-Class A Comprehensive Teaching Hospital* (Southern Medical University, 2013).

[21] Wicker S, Rabenau HF (2020). Occupational exposures to bloodborne viruses among German dental professionals and students in a clinical setting. Int. Arch. Occup. Environ. Health.

[22] DeGirolamo KM, Courtemanche DJ, Hill WD (2013). Use of safety scalpels and other safety practices to reduce sharps injury in the operating room: What is the evidence?. Can. J. Surg..

[23] Reddy VK, Lavoie MC, Verbeek JH (2017). Devices for preventing percutaneous exposure injuries caused by needles in healthcare personnel (review). Cochrane Database Syst. Rev..

